# Successful Retirement Planning for Endocrinologists

**DOI:** 10.1210/jendso/bvae166

**Published:** 2024-10-09

**Authors:** Carolyn B Becker, Karen E Friday, Alan D Rogol, William F Carroll, Richard J Santen

**Affiliations:** Division of Endocrinology, Brigham and Women's Hospital, Harvard Medical School, Boston, MA 02115, USA; 1004 Philip Street, New Orleans, LA 70130, USA; Division of Pediatric Diabetes and Endocrinology, University of Virginia, Charlottesville, VA 22908, USA; 6919 Spring Valley Road, Dallas, TX 75254, USA; Division of Endocrinology and Metabolism, University of Virginia, Charlottesville, VA 22908, USA

**Keywords:** retirement, planning, volunteering, mentoring teaching, giving back

## Abstract

The Endocrine Society formally addressed the issue of retirement for its members for the first time in a Workshop held on June 4 at ENDO 2024 in Boston, Massachusetts. Preparation for the workshop included 4 steps: (1) completion of a survey; (2) advice from a retirement expert; (3) extensive literature review; and (4) multiple pre-workshop discussions among the presenters. The survey found that retired endocrinologists are involved in a wide variety of professional and nonprofessional activities. The retirement expert and the literature review outlined several concepts underlying a successful retirement and the questions and decisions needed during the planning process. The presenters described several illustrative examples of retirement activities. A “Piece of My Mind” essay in the *Journal of the American Medical Association* written by the moderator (C.B.) expressed ethical considerations made feasible by the independence of her retirement status. The first presenter (A.R.) noted that the time available during retirement allowed mentorship, teaching in foreign countries and other institutions, and participation on international committees. The second speaker (K.F.) commented that expertise gained during practice of Endocrinology can be used for expert legal, pharmaceutical, and financial opinions. She also noted that volunteering for professional or nonprofessional groups provides an avenue for “giving back” to others. The final presenter (R.S.) stated that retirement provides an opportunity to embark on new clinical endeavors, such as managing patients via telemedicine in rural underserved areas. In summary, retirement is an important phase of a career and can be highly rewarding and enjoyable.

Over the past 7 decades, the average lifespan for men and women in the United States has increased from age 67 to 79 years [1]. As the average age of retirement now approximates 65 years, individuals currently live for nearly 14 years after retirement [2]. During the early retirement years, individuals are more likely to be in good health, of sound mind, and have a substantial reservoir of energy. This is a time when one can contribute to society, take up completely new endeavors, enjoy hobbies and activities, and spend more time with family and friends. A significant challenge is to determine one's purpose in life during the retirement years and to develop a structure that profitably and enjoyably invests that time. Individuals who are retiring have gained a wealth of wisdom and experience over their working careers and possess a number of important core competencies. Not utilizing those during the retirement years represents an unnecessary waste of talent.

The Endocrine Society (ES) has previously devoted substantial resources to facilitate early career development but has not until now considered the retirement phase. Accordingly, one of the authors (R.J.S.) submitted a proposal to the Annual Meeting Steering Committee to hold a workshop on retirement that would provide useful information for those planning retirement or already retired. The idea was accepted, and the workshop was held at ENDO 2024 in Boston. In this manuscript, we highlight the concepts and conclusions arrived at by reviewing the appropriate literature and the presentations and discussion involved in this workshop.

## Methods for Workshop Planning

Several steps were taken to plan for this event.

**Step 1.** We developed a survey and contacted individuals in the ES directory listed as retired and asked them to complete it.

**Step 2**. We requested advice from a recognized retirement expert, our co-author, Dr. William F. Carroll Jr., who had developed a program called “Skydiving Into Retirement” for members of the American Chemical Society [3].

**Step 3.** We reviewed published literature that discussed the various components of retirement applicable to a wide range of retirees [2-6].

**Step 4**. We participated in extensive discussions with the moderator (C.B.) and 3 speakers (A.R., K.F., and R.S.) selected for the workshop.

## Results

### Step 1: Survey Results

**Figure 1.**
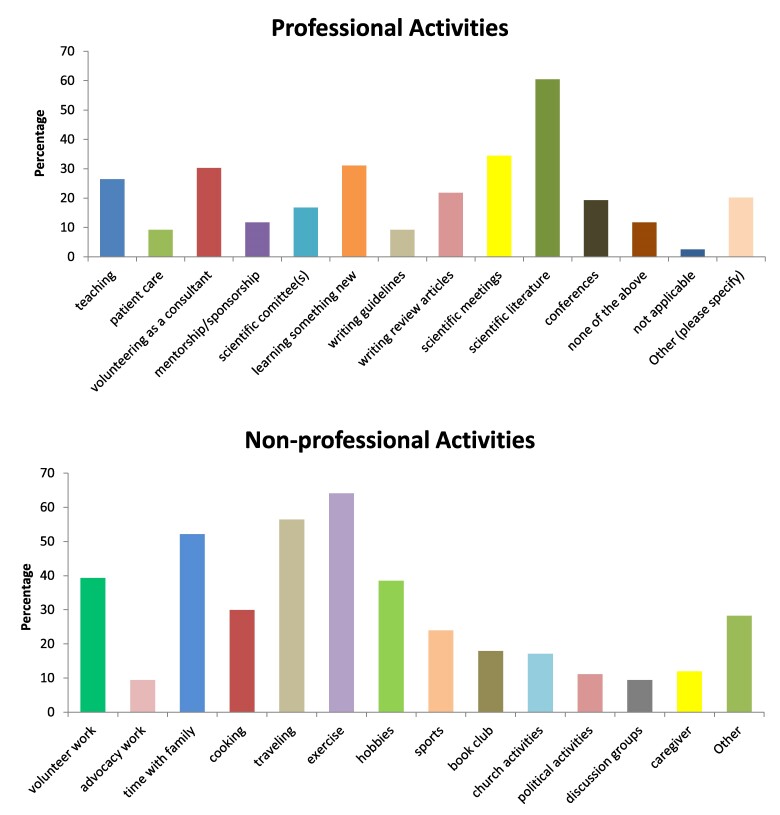
Activities during retirement. Top panel: professional activities (to reduce the extensive descriptions on the figure, a full description of categories is listed here in the order in the figure): teaching; patient care, volunteering or working as a medical or research consultant; mentorship/sponsorship of young people in medicine or in science; scientific committee(s); learning something completely new to you; writing guidelines; writing review articles; attending scientific meetings; keeping up with scientific literature; participating in local clinical or research conferences; none of the above; not applicable; other. Bottom panel: nonprofessional activities (full description of categories in order): volunteer work in community; advocacy work; spending more time with family; cooking; traveling; exercise; hobbies; sports; book club; church activities; discussion groups; care giver (child, elderly, or other); other.

A total of 119 ES members completed the on-line survey. The majority (75%) were fully retired and another 20% were semi-retired. Eighty percent of the respondents identified as male and nearly 50% of all respondents had worked as clinical practitioners. The remainder identified as educators, clinical researchers, or basic researchers. The 3 top concerns about retirement were: 1) loss of professional and social connections; 2) worries about physical health; and 3) loss of professional identity. Retirees have engaged in a variety of professional and nonprofessional activities as shown in Fig. 1. When asked what the retirees would like the ES to offer to support retirees, suggestions included discounted or free ES services (such as membership fees, meeting registration, journal fees, courses), a mentorship match for retirees and young mentees, a volunteer page on the ES website offering opportunities, and periodic symposia, workshops, receptions, or discussion groups for those thinking about or already retired.

### Step 2: Retirement Expert Advice

Dr. William Carroll Jr., a Certified Professional Retirement Coach, provided further advice. He said that for the first 3 years of his retirement, he experienced depression. In introducing himself to others, he felt ashamed to say that he was retired. He indicated that when he had been formerly employed, he had structured no boundaries between his work and himself, and at the end of his canonical career he was unsure of his identity. His key insight—perhaps obvious in retrospect—was that he was not the *only* person who experienced this jarring transition [7]. Retired men (or women) are significantly more likely to experience depression than those who are employed, and the retirement transition is approximately half as stressful as losing a spouse [7].

The question is “what to do about it”? Dr. Carroll began to develop plans and structure for a workshop on the retirement transition and this ultimately resulted in his focus on some educational aspects of retirement. The 3-hour workshop called “Skydiving Into Retirement” is provided in-person and free of charge to American Chemical Society members at their semi-annual national meetings and by appointment remotely, otherwise [7]. The workshop contains both didactic and hands-on exercises. While the workshop is not intended to provide financial advice, Dr. Carroll recommends that retirees with financial questions engage a professional to act as a fiduciary.

What about the transition from working to retirement? Dr. Carroll posed a series of questions. How do you know it is time to retire? Should you do this cold turkey or wind down? Should you consider half-time retirement for a period of time before full retirement? What are you going to do during retirement? What would you like to do? What is its value? What things do you not like to do? He commented that one should inventory core competencies and recognize that all ES members are literate and have been successful during their careers—attributes that not everyone can claim. One has value!

Carroll's workshop emphasizes 3 aspects: *Identity*, *Structure*, and *Purpose* and these 3 aspects should be adapted by individuals. While there are no set rules, understanding how you view yourself as you transition from a full-time career is critical. It is most important to decide how much time you devote to specific activities and to find the right balance between structured and unstructured time. Finally, understanding why you do what you do and for whom you do it can bring peace of mind.

How much time should one devote to various activities during retirement? A pitfall is to get trapped into actually working full-time, particularly if your intention was to work only part-time. On the other hand, some structure is necessary. Working professionals likely spend upwards of 2000 hours per year in activity structured by “work.” For many people, retirement is the first opportunity they have had to personally structure such a large block of time.

Carroll suggests active management, and a mix of structure and opportunity. Each day is different and interesting only because you make it so. Make some commitments to groups, who expect you to be there and will miss you if you are not. This can be as simple as a golf group, a book club, or various civic or professional activities. The key is to provide yourself with activities and social contact; he notes that the less you do, the easier it is to do even less.

There is a dauntingly large literature on how to structure retirement [2, 4-9]. Much of it focuses on making the most of the first years because they will tend to be the healthiest and most active. Some have characterized retirement into 3 phases: the first is *go-go*, the second is *slow-go*, and the third is *no-go*. A good deal of this depends on one's health and temperament. One has to plan retirement activities based on what is physically possible. Less obvious are decisions about place: Does your community and your dwelling lend itself to your physical and social needs? Not everyone needs to move to a new place or downsize, but some people do, and the number of alternatives is large. Decisions about place should also be active decisions.

In the end, Dr. Carroll reiterated these 3 concepts: *Identity:* When you were working as a professional, you had a clear idea of who you were, what you did, and why you did it. Who are you now? Have you learned to like that person? When someone says, “Tell me a little about yourself,” what do you say? *Structure:* Have you built in enough regular physical—yes, appropriate exercise—and social interactions to make each day an experience? *Purpose:* Is there a central organizing theme to your activities?

### Step 3: Literature Review

In a recent *Harvard Business Review*, Dychtwald, Morrison, and Terveer wrote an article titled “Redesigning Retirement” focusing on the business workforce, but also providing important insight for endocrinologists [2]. More than 10 million Americans who are 65 years and older are currently employed, and that number is projected to rise to nearly 15 million by 2032 [10, 11]. Twenty-seven percent of Americans ages 65-70 are working or looking for work [2, 10]. Of these individuals 83% say that **“feeling useful is more important to them than feeling youthful”** [2]. Many “working retirees” are happy to have part-time jobs and familiar roles [2, 7]. Others want to try something entirely different, sometimes related to an avocation put aside earlier in their lives or just something new. Older, more experienced individuals have substantial value. They can serve as role models reflecting resilience and emotional maturity to their younger colleagues [2]. These individuals can also mentor younger individuals in an ongoing way, teach them in formal training programs and informal sessions, and coach them, often as they transition into new roles.

Research finds that most older workers are curious and eager to learn [2]. More experienced adults tend to have higher levels of **“crystallized intelligence”** which enables them to make connections and informed decisions, whereas less experienced workers tend to have higher levels of “**fluid intelligence”** which involves abstract thinking and new ideas [2]. The Dychtwald et al article stated that 68% of workers aged 50 or older would like the opportunity to move into retirement gradually [2]. Two-thirds of older Americans now view retirement not as a time of pure rest and relaxation, but as a new chapter in life, with plenty of room for new ambitions and renewed purpose [2]. One method is to permit them to apply their skills in new ways, learning new things, and making new contacts.

The key components to successful retirement are timing and flexibility [2]. The COVID-19 epidemic experience demonstrated that working virtually with use of meeting platforms, such as ZOOM and Teams, could allow productive work without travel to the office [12]. Dychtwald et al comment that older employees appreciate the benefits of working remotely [2]. These authors also state that “our research finds that older employees appreciate the social connections of the workplace and so tend to be happy to work on site when necessary.”

**A win-win:** Dychtwald et al first explored these challenges and opportunities in the *Harvard Business Review* exactly 20 years ago, in an article titled “It Is Time to Retire Retirement.” The authors see progress over this time period [2, 11]. Today, more older people want or need to work longer, and more organizations than ever require their help. In this new age of aging, the strategies and initiatives the authors have described present a clear win-win. When older adults stay active and are engaged, it is often good for them and their families, for employers, the younger, full-time workers, the economy, and for society at large. However, the wise person knows when to step down.

Lawrence Robinson and Melinda Smith wrote about aging issues in an article titled “Adjusting to Retirement: Handling Depression, Stress and Anxiety” [7]. As shown in Table 1, they comment on common challenges of retirement. These authors suggest 4 tips for adjusting to retirement and particularly managing psychological issues (Table 2). One emphasis is on volunteering, either in the profession or in a nonprofessional area. An important issue is to be receptive and supportive to the needs of your life partner in planning retirement. Kathleen Coxwell identifies 9 topics to tackle to survive retirement with your spouse [13]. She comments that if struggling to get on the same page with your spouse, an advisor may help.

**Table 1.** Common challenges of retirement

Struggling to “switch off” from work mode and relax, especially in the early weeks or months of retirement
Feeling anxious at having more time on your hands, but less money to spend
Finding it difficult to fill the extra hours you now have with meaningful activity
Losing your identity. If you are no longer a doctor, teacher, designer, salesperson, electrician, or driver, for example, who are you?
Feeling depressed and isolated without the social interaction of being around your co-workers
Experiencing a decline in how useful, important, or self-confident you feel
Adjusting your routine or maintaining your independence now that you’re at home with your spouse or significant other during the day
Some retirees even feel guilty receiving money from a pension without directly working for it

Table adapted from Robinson and Smith [7].

**Table 2.** Tips to help ensure a successful retirement

**Tip 1**	Embrace change and adjust your attitude, build resilience, accept the things that you cannot change, redefine your identity, set new goals, seek social support, strengthen your social network, enroll in a retirement transitions program, and join a peer support group.
**Tip 2**	Find new purpose and meaning. This includes consideration that retirement may not have to be all-or-nothing and one can gradually transition into full-time retirement rather than to jump right in. Find part-time work after retirement. Volunteer and donate your time and effort to a cause that is important. Nurture hobbies and interests. Learn something new. Get a pet.
**Tip 3**	Manage retirement depression, stress, and anxiety. Take specific actions, such as adopt a relaxation practice, get active, practice gratitude, spend time in nature, break the worry habit.
**Tip 4**	Look after your health. Get enough quality sleep, eat a healthy diet, watch how much you drink, keep challenging your brain, and add structure to your days.

Several articles discussed the issue of physician burnout and early retirement [9, 12, 14-19]. This is a common phenomenon among physicians in general and applies to endocrinologists as well [20]. With early retirement, one has to consider how to utilize many more years without structured working. These concepts discussed for retirement generally apply specifically to early retirees.

### Step 4: Workshop Scope

The moderator and speakers met to consider what would be the key components of the workshop. These included: (1) presentation of personal examples of both professional and nonprofessional activities undertaken; (2) our judgment of the value of these experiences; and (3) a series of questions to the participants in the workshop to facilitate wider discussion.

## Presentations at Workshop

Moderator Carolyn Becker reviewed the results of the survey data and the varied professional and nonprofessional activities reported (Fig. 1). She noted that multiple suggestions about how the ES might implement programs to aid retired endocrinologists were included in responses to the Survey; Supplementary Appendix [21]. Dr. Becker then shared her varied experiences as a retired endocrinologist, including writing a “Piece of My Mind” essay for the *Journal of the American Medical Association* on the interactions between Academia and industry [22]; attending the Brigham and Women's Hospital Endocrine Case Conferences; volunteering to take care of patients via telemedicine with a company called Maven, Inc; extensive traveling; and a number of other activities.

Alan Rogol emphasized the important role of retired endocrinologists as mentors. He reviewed the history of the man named Mentor from the *Illiad* by Homer as the origin of the word mentorship. He commented that one of the most important activities that retired endocrinologists can do is to mentor the subsequent generations of endocrinologists.

Dr. Rogol described some of the differences between mentoring, a learning relationship generally focused on long-term career development, and coaching, assisting people with their current performance. Before describing his personal activities in retirement, he outlined some broad categories of activities in which retirees might partake:

- Career-related
  - Teaching
  - Patient-related
  - Consulting
  - Review of scientific and clinical materials (referee)
  - Writing scientific and clinical reports
- New activities, unrelated to their former career
- Exercise/health-related
- Family/friends
- Other

Dr. Rogol concluded his portion of the workshop by describing some of the highlights of his activities during retirement as shown in Table 3.

**Table 3.** Examples of retirement activities undertaken by Dr. Alan Rogol

Clinically related Staffing a pediatric clinic in Tovar, Haiti Teaching pediatric endocrinology to African pediatricians at both of the European Society for Pediatric Endocrinology (ESPE) Training Centers in Africa Nairobi, Kenya (East Africa) Lagos, Nigeria (West Africa) Teaching (part-time) Riley Hospital for Children (Indianapolis, IN) and the University of Virginia (Charlottesville, VA) Visiting professor at several academic institutions, nationally and internationally Science and clinical science-related Reviewing scientific reports for clinical and scientific journals Reviewing therapeutic use exemptions for banned drugs by elite athletes (anti-doping) Consulting, mainly for Pharma Data safety and management boards Personal Daily exercise both endurance and resistance Maintaining contact with about a dozen retired professors from the Department of Pediatrics at the University of Virginia—at a minimum we join for breakfast twice monthly Helping wife with her crafts hobby/business at craft fairs d. Reading for pleasure

Karen Friday spoke about her professional and nonprofessional activities during retirement (Table 4). Dr. Friday emphasized the variety of activities that she had engaged in and the many opportunities that are available to retirees. She also identified resources available to help individuals search for volunteer or part-time work opportunities. Volunteer opportunities have been available through the American College of Physicians (ACP; current membership required to access the ACP volunteer website). Medscape recently discussed physician volunteer opportunities [23]. Resources for part-time, remote, or nonclinical work opportunities are available at Indeed.com and the SEAK Non-Clinical Careers for Physicians annual conference. Many health professionals have found colleagues or LinkedIn to be valuable resources for networking or to find new opportunities.

**Table 4.** Varied activities described by Dr. Karen Friday

**Clinical**	Locums
	Endocrine Society DocMatter Community
	Second opinions
	Prior authorization reviews
**Academic**	Endocrine teaching conferences
	Lecturer society meetings
	Abstract reviews Endocrine Society/nutrition
	Manuscript reviews
	Medical student/resident mentoring for research
**Research**	Consultant local clinical trials group
	Consultant pharmaceutical companies
**Endocrine expert witness**	Medical record review for legal teams
	Literature review for legal cases
	Expert depositions
**Personal-Nonprofessional**	Renovation historic home in New Orleans
	Gardening
	Photography
	Cooking
	Glassblowing
**Medical device design**	Designed artistic sensory monofilament from wood
	Collaboration with local artisan/woodworker

Richard Santen indicated that he had chosen a very specific retirement project and the reasons behind this decision. This activity included caring for patients with diabetes in rural, financially challenged areas via telemedicine. He commented that a major gap in the United States has been the limited number of endocrinologists available to see patients in these areas [24]. With the use of telemedicine, endocrinologists can take care of these patients with diabetes remotely. The Federally Qualified Community Health Care system consists of 1400 clinics in the USA and is supported by 5.7 billion dollars of federal government support annually [24]. These clinics lack access to specialists, and the providers welcome the assistance of endocrinologists working part-time and remotely.

Dr. Santen described how he established a relationship with 6 Federally Qualified Community Health Centers in rural southwest Virginia. The plan was to reduce glucose levels, and together with the nurse educator, instruct in weight loss, exercise, and life-style management. After an initial evaluation via telemedicine, he then has patients call him once per week to report glucose levels or review continuous glucose monitor (CGM) data.

He has now completed and published the evaluation of 136 patients over a period of 6 years [24]. The hemoglobin A1C levels have fallen substantially from 10.3 ± 1.94% to 7.8 ± 1.31%. In analyzing a subset of patients at the time of discharge from his program and 6 to 18 months later, the average hemoglobin A1C did not decline any further: 7.85 ± 1.54% at discharge and 7.92 ± 1.07% on follow-up [24]. He noted that this retirement project has been enjoyable and provides the ability to manage patients without the administrative hassle usually involved in taking care of patients in a highly organized University Hospital.

## Discussion

The workshop on retirement for endocrinologists at ENDO 2024 represented the first time that the ES has considered this important aspect of career development. During the preparation for this event, a detailed literature review revealed several important concepts and considerations [2, 4-8, 10, 11, 25]. Retirement can be a time of continued professional fulfillment, but also redirection toward interests and activities that were not possible due to previous time constraints. Establishment of the purpose for one's retirement is paramount. A key concept is the possibility of “giving back” to the profession based on one's experience. Another is that without careful retirement planning and implementation, one can become depressed or anxious and lose a sense of one's identity. Several challenges face the endocrinologist planning to retire or in the earlier phases of retirement. Approximately 2000 hours per year are available to occupy upon retirement and one needs to implement a strategy to fill that time with purpose and enjoyment.

The literature review emphasized making decisions about purpose, one's identity, and careful planning of activities and utilization of one's time. Without carefully considering these aspects, early retirement can be daunting, with the onset of depression and anxiety. With careful planning, retirement can be part of a broader and deeply significant transformation in the shape of one's life. Of note, 71% of Americans aged 65 years or older say that the best time of their lives is not in the past but right now or still in front of them [2]. This has a powerful implication: if retirement means completely ceasing work and devoting 2 or more decades to complete leisure, it is increasingly impractical, unappealing, and obsolete. Comments from the presenters and attendees also focused on the importance of giving back to the profession based on one's experience, wisdom, and talents. Wisdom is an important commodity, and it is a shame to have it wasted during retirement [2, 11].

The survey of retired endocrinologist revealed multiple activities in the professional realm that were reported by retired ES members (Fig. 1, top panel). The lessons learned from the literature and the survey questionnaire are that retirement can be enjoyable, productive for one's profession, and representative of a new experience in one's life. The questions arising during the Workshop, the review of the activities of retirees during a survey, and the specific activities of the presenters, taken together, have provided the landscape upon which the ES can build in the future with respect to retirees (see Supplementary Appendix). The attendees at the workshop were asked about their opinion about continuing sessions at the annual meetings of the ES and the majority endorsed supporting continued programs for retirees initiated by the ES. While the workshop itself did not discuss a practical roadmap for retirees, each of the aspects of retirement discussed both at the workshop and by the literature later guided us in this manuscript to provide a roadmap (Table 5).

**Table 5.** Road map of steps to take regarding retirement

Decide when to retire or gradually retire
Decide on what will be your primary purpose during retirement
Obtain a financial planner and implement all financial and insurance steps suggested
Establish a timeline for your retirement or gradual retirement
Notify all stakeholders and patients about your specific plans and timeline
Decide on the professional activities that you might wish to continue with and organize
Decide on the nonprofessional activities that you might wish to do and organize
Set up a plan which identifies activities during each day
Establish a plan for maintenance of your health
Decide how and when to increase your family interactions
Discuss all in detail with your spouse, partner, or significant other
Make plans to update your medical license and continuing education activities, if needed
Consider the issues of downsizing your home and planning for long-term health care
Re-update your will, if needed
Consider joining professional or nonprofessional retirement groups and groups that might benefit from your expertise—science museums, homeowner groups, and a myriad of others

The authors placed emphasis on the fact that Americans are living longer than they did in previous generations and that those 65 to 75 years old probably have the energy and motivation that was similar to those in their 50s and 60s just a couple of decades ago. Much of this relates to the fact that Americans are now much healthier than they had been 2 decades ago and live longer. At age 65 they can look forward to at least 20 more years on average before passing. These years can be highly productive and enjoyable if the appropriate steps are taken.

An interesting term was encountered, namely *working retirees*, and it was noted that these individuals are happy to have part-time jobs in familiar roles, often in their former settings. At the same time, others want to try something entirely different, related to an avocation they put aside early in their lives. Little emphasis was placed on financial issues during the workshop. Most endocrinologists have invested in financial retirement programs or have pensions that do cover their retirement years. However, it was noted in the literature that if older people have more years to enjoy purposely, they also have more years to fund [2]. The article in the *Harvard Business Review* emphasizes what older, more experienced workers bring to the table, and this is well-documented yet highly underappreciated [2]. These individuals have knowledge and skills, both functional and organizational. They offer commitment, loyalty, and engagement—valuable traits often in decline these days. They consistently served as mentors and role models and help to guide their younger colleagues.

The authors recognize that the survey utilized for obtaining preliminary information was not comprehensive nor designed by experts in survey development. We recognize the need for additional information about retirees that can be accomplished by a more rigorously designed survey and dissemination to a wider group of endocrinologists planning to retire or already retired.

In summary, retirement is an important phase of the life of an endocrinologist and can be highly rewarding and enjoyable provided the proper planning precedes the actual retirement process. Several steps in this process are needed, both financial, organizational, conceptual, and implementable. A valuable activity concerning planning retirement is to review retirement literature and follow various steps that have been taken by others who have achieved a highly successful retirement.

## Disclosures

No disclosures.

## Data Availability

All data can be accessed via references.

## Abbreviation

ES: Endocrine Society
